# Alveolar gas exchange and tissue oxygenation during incremental treadmill exercise, and their associations with blood O_2_ carrying capacity

**DOI:** 10.3389/fphys.2012.00265

**Published:** 2012-07-11

**Authors:** Antti-Pekka E. Rissanen, Heikki O. Tikkanen, Anne S. Koponen, Jyrki M. Aho, Harriet Hägglund, Harri Lindholm, Juha E. Peltonen

**Affiliations:** ^1^Department of Sports and Exercise Medicine, Institute of Clinical Medicine, University of HelsinkiHelsinki, Finland; ^2^Clinic for Sports and Exercise Medicine, Foundation for Sports and Exercise MedicineHelsinki, Finland; ^3^Centre of Excellence for Health and Work Ability, Finnish Institute of Occupational HealthHelsinki, Finland

**Keywords:** near-infrared spectroscopy, oxygenation, CO-rebreathing method, blood oxygen carrying capacity, treadmill exercise

## Abstract

The magnitude and timing of oxygenation responses in highly active leg muscle, less active arm muscle, and cerebral tissue, have not been studied with simultaneous alveolar gas exchange measurement during incremental treadmill exercise. Nor is it known, if blood O_2_ carrying capacity affects the tissue-specific oxygenation responses. Thus, we investigated alveolar gas exchange and tissue (m. vastus lateralis, m. biceps brachii, cerebral cortex) oxygenation during incremental treadmill exercise until volitional fatigue, and their associations with blood O_2_ carrying capacity in 22 healthy men. Alveolar gas exchange was measured, and near-infrared spectroscopy (NIRS) was used to monitor relative concentration changes in oxy- (Δ[O_2_Hb]), deoxy- (Δ[HHb]) and total hemoglobin (Δ[tHb]), and tissue saturation index (TSI). NIRS inflection points (NIP), reflecting changes in tissue-specific oxygenation, were determined and their coincidence with ventilatory thresholds [anaerobic threshold (AT), respiratory compensation point (RC); V-slope method] was examined. Blood O_2_ carrying capacity [total hemoglobin mass (tHb-mass)] was determined with the CO-rebreathing method. In all tissues, NIPs coincided with AT, whereas RC was followed by NIPs. High tHb-mass associated with leg muscle deoxygenation at peak exercise (e.g., Δ[HHb] from baseline walking to peak exercise vs. tHb-mass: *r* = 0.64, *p* < 0.01), but not with arm muscle- or cerebral deoxygenation. In conclusion, regional tissue oxygenation was characterized by inflection points, and tissue oxygenation in relation to alveolar gas exchange during incremental treadmill exercise resembled previous findings made during incremental cycling. It was also found out, that O_2_ delivery to less active m. biceps brachii may be limited by an accelerated increase in ventilation at high running intensities. In addition, high capacity for blood O_2_ carrying was associated with a high level of m. vastus lateralis deoxygenation at peak exercise.

## Introduction

Cardiopulmonary responses to incremental exercise are controlled through a complex combination of feedback- and feedforward mechanisms including metabolic changes within the active muscle mass and cerebral tissue (Turner, 1991; Ogoh and Ainslie, 2009). However, the role of less active muscles has remained sparsely studied. Changes in local oxygenation of muscle- and cerebral tissue can be estimated continuously and non-invasively by near-infrared spectroscopy (NIRS) (Boushel et al., 2001). The NIRS signal obtained during exercise can be employed to reflect the relationship between local O_2_ delivery and utilization (Δ[O_2_Hb], Δ[HHb], TSI), or changes in tissue blood volume (Δ[tHb]) at the site of O_2_ exchange (Boushel et al., 2001; DeLorey et al., 2003).

During incremental treadmill exercise, oxygenation in m. vastus lateralis remains relatively constant during walking, but decreases during running as a result of increased local O_2_ extraction and utilization due to increasing muscle work (Hiroyuki et al., 2002; Lee et al., 2011). Arm muscle oxygenation has not been studied during incremental treadmill exercise, but during incremental cycling, an initial moderate decrease in oxygenation followed by a rapid decrease during more severe exercise has been reported (Ogata et al., 2007; Peltonen et al., 2012). Cerebral tissue oxygenation has a quadratic response to incremental exercise; it increases from low-to-hard intensities, followed by a plateau or decline toward baseline during severe exercise (Nielsen et al., 1999; Rooks et al., 2010). Concerning incremental treadmill exercise, NIRS trends have not been compared with alterations in alveolar gas exchange. However, an exaggerated decline in active muscle oxygenation coinciding with anaerobic threshold (AT) (e.g., Bhambhani et al., 1997), an exaggerated decline in less active muscle oxygenation coupling with increase in the amount of hyperventilation (Ogata et al., 2007; Peltonen et al., 2012), and a decrease in cerebral oxygenation following respiratory compensation point (RC) (e.g., Bhambhani et al., 2007), have been demonstrated during incremental cycling.

The O_2_ delivery of the blood and the O_2_ utilization of the whole body determine peak pulmonary O_2_ uptake (${\dot{\text{V}}\text{O}}_{\text{2peak}}$), which is a key determinant of aerobic capacity. Peak pulmonary O_2_ uptake has a strong association with tHb-mass which together with blood volume determines hemoglobin concentration and blood O_2_ carrying capacity (Schmidt and Prommer, 2010). Herewith, tHb-mass is a vital part of O_2_ delivery. However, evidence of associations between ${\dot{\text{V}}\text{O}}_{\text{2peak}}$ and tissue oxygenation during exercise is limited, and the effect of blood O_2_ carrying capacity on oxygenation is unknown. During submaximal cycling intensities, the level of m. vastus lateralis deoxygenation is lower in individuals with higher ${\dot{\text{V}}\text{O}}_{\text{2peak}}$ (Costes et al., 2001; Boone et al., 2009). At peak exercise, the findings are controversial; in some studies, associations between the extent of active muscle deoxygenation and ${\dot{\text{V}}\text{O}}_{\text{2peak}}$ have not been found (Neary et al., 2002; Boone et al., 2009), whereas Bae et al. (1996) have observed a positive association. Hogan et al. (1999) have reported a similar intracellular metabolic environment attained at exhaustion among the varied fraction of inspired O_2_. In cerebral tissue, a plateau in Δ[O_2_Hb], concurrent with increasing Δ[HHb] and Δ[tHb], has been reviewed in individuals with higher ${\dot{\text{V}}\text{O}}_{\text{2peak}}$ at high exercise intensities (Rooks et al., 2010).

In the present study, we: (1) measured alveolar gas exchange and tissue oxygenation in leg and arm muscle, and in cerebral tissue, simultaneously during incremental treadmill exercise; (2) compared timings of alterations in tissue oxygenation and alveolar gas exchange; and (3) examined, if blood O_2_ carrying capacity affects tissue-specific oxygenation responses. We hypothesized that: (1) regional tissue oxygenation would be characterized by tissue-specific inflection points; (2) an exaggerated decline in leg muscle oxygenation coinciding with AT, an exaggerated decline in arm muscle oxygenation coupling with increase in the amount of hyperventilation, and a decrease in cerebral oxygenation following RC, would be manifested; and (3) blood O_2_ carrying capacity would have a negative association with the extent of tissue deoxygenation during submaximal exercise but no association at peak exercise.

## Materials and methods

### Subjects

Twenty-two healthy male volunteers gave written informed consent to participate in the study. The subjects were non-smoking, free of medication, and had no history of cardiovascular, respiratory, endocrinological, musculoskeletal, or neurological diseases. The anthropometric and spirometry data, and ${\dot{\text{V}}\text{O}}_{\text{2peak}}$ of the subjects are shown in Table 1. The reference values of the spirometry have been published for the Finnish population (Viljanen et al., 1982). The study was approved by the Ethics Committee of the Hospital District of Helsinki and Uusimaa, Helsinki, Finland, and conforms to the Declaration of Helsinki.

**Table 1:** **Anthropometric and spirometry data, and peak pulmonary O_2_ uptake of the subjects (*n* = 22)**.

Age (years)	29 ± 6
Weight (kg)	79.3 ± 8.5
Height (cm)	180 ± 8
BMI (kg/m^2^)	24.5 ± 2.1
Fat %	13.5 ± 4.6
FVC (L)	6.1 ± 0.7
FVC (% of reference value)	108 ± 7
FEV_1_ (L)	4.8 ± 0.6
FEV_1_ (% of reference value)	101 ± 11
FEV_1_/FVC (%)	78.9 ± 6.8
${\dot{\text{V}}\text{O}}_{\text{2peak}}$ (ml · kg^−1^ · min^−1^)	50 ± 6

Values are means ± SD. BMI, body mass index; FVC, forced vital capacity; FEV_1_, forced expiratory volume in one second; ${\dot{\text{V}}\text{O}}_{\text{2peak}}$, peak pulmonary oxygen uptake.

### Experimental sequence and exercise protocol

The experimental protocol consisted of pre-exercise measurements and a cardiopulmonary exercise test on a treadmill. Subjects reported to the laboratory two to three hours after a meal, and after abstaining from physical exercise for at least 12 h and alcohol for at least 24 h. All subjects completed a preliminary data form concerning personal health, medical history, and smoking habits. The subject's height was measured. The body composition was determined by the bioimpedance method (InBody 720, Biospace Co., Ltd., Seoul, South-Korea). Pre-exercise measurements at rest also included blood pressure, a 12-lead ECG, and flow-volume spirometry (Medikro Spiro 2000, Medikro, Kuopio, Finland). A physician examined the subject to ensure suitability for exercise testing.

Each subject performed a cardiopulmonary exercise test on a treadmill (Juoksumatto OJK-1, Telineyhtymä, Kotka, Finland). The step incremental protocol was preceded by 3 min rest while the subjects stood on the treadmill, followed by 5 min baseline walking at a speed of 5 km · h^−1^. The incremental exercise (1 km · h^−1^ · 3 min^−1^) was then initiated with a speed of 8 km · h^−1^, and the subjects continued exercising until volitional fatigue. Throughout the test, the incline of the treadmill was 0.5°, mimicking air resistance during outdoor running.

### Cardiopulmonary responses

Heart rate and the electrical activity of the heart were monitored by ECG throughout the test. Arterial O_2_ saturation (SpO_2_%) was monitored by finger tip pulse oximetry (Nonin 9600, Nonin Medical, Inc., Plymouth, USA). Breath-by-breath ventilation was measured by a low-resistance turbine (Triple V, Jaeger Mijnhardt, Bunnik, The Netherlands). The turbine was calibrated using a syringe of known volume (3.00 L, Hans Rudolph Inc., Kansas City, MO, USA). Expired and inspired gases were sampled continuously at the mouth and analyzed for concentrations of O_2_, CO_2_, N_2_-, and Ar by mass spectrometry (AMIS 2000, Innovision A/S, Odense, Denmark) after calibration with precision analyzed gas mixtures. Breath-by-breath respiratory data were collected in a raw-data mode. The raw data were transferred to a computer where gas delays were determined for each breath in order to align concentrations with volume data, and to build a profile of each breath. Breath-by-breath alveolar gas exchange was then calculated with the AMIS algorithms, slightly modified from the original algorithms presented by Beaver et al. (1981). A moving average of the individual test data was calculated over 5 s periods to lessen some of the inherent breath-by-breath variability, and interpolated to obtain values second by second. The ventilatory thresholds (i.e., AT and RC point) were determined using the V-slope method (Beaver et al., 1986). Values of pulmonary O_2_ uptake were handled as both absolute (${\dot{\text{V}}\text{O}}_{\text{2}}$) and relative (%${\dot{\text{V}}\text{O}}_{\text{2}}\text{R}$ = percentage value of ${\dot{\text{V}}\text{O}}_{\text{2}}$ reserve; ${\dot{\text{V}}\text{O}}_{\text{2}}$ reserve is a difference between ${\dot{\text{V}}\text{O}}_{\text{2peak}}$, and ${\dot{\text{V}}\text{O}}_{\text{2}}$ at rest).

### Regional cerebral and muscle oxygenation

#### NIRS measurements

Local oxygenation profiles were monitored simultaneously from leg muscle, arm muscle, and cerebral tissue using a continuous wave NIRS system (Oxymon Mk III Near-Infrared Spectrophotometer, Artinis Medical Systems, Zetten, The Netherlands). For local leg muscle oxygenation profiles, the transmitting and receiving optodes were placed over the vastus lateralis muscle of the right leg at mid-thigh level and parallel to the long axis of the muscle. Since the activity of m. vastus lateralis increases during running (Guidetti et al., 1996), this muscle is suitable for examining exercise-induced changes in leg muscle oxygenation. To monitor less active arm muscle during running, the optodes were positioned longitudinally on the biceps brachii muscle of the right arm above the elbow joint and lateral to middle line. For cerebral monitoring, the optodes were placed over the right frontal cortex, about 2 cm above the right eyebrow and as laterally as possible to the longitudinal fissure of cerebrum. The prefrontal association cortex occupies most of the rostral part of the frontal cerebral lobe. This site has been linked to the planning of voluntary movement (Sahyoun et al., 2004), and it may contribute to an integrative decision to stop exercising. For both muscle and cerebral measurements, the optodes were housed in an optically dense plastic holder which was attached to the skin using double-sided adhesive tape. For the cerebral optode holder, a special headband was used. The setup helped to minimize the intrusion of extra outside light and the loss of transmitted near-infrared light from the field of interrogation and to ensure the fixed positions of the optodes. The inter-optode distances were chosen to be between 35 and 50 mm so that a good signal quality was reached before starting the measurements. A sampling frequency of 50 Hz was used for collecting NIRS data.

Each NIRS probe consisted of one receiver and three transmitters operating at wavelengths of 765 and 860 nm. The theory of NIRS and its use in exercise measurements have been described in detail elsewhere (Boushel et al., 2001). Briefly, the intensity of incident and transmitted light was recorded continuously and, along with the specific optical pathlength and extinction coefficients, used for on-line estimation and display of concentration changes (ΔμM) from the resting baseline of oxyhemoglobin (Δ[O_2_Hb]), deoxyhemoglobin (Δ[HHb]), and total hemoglobin (Δ[tHb] = Δ[O_2_Hb] + Δ[HHb]). The values used for the differential pathlength factor (DPF) were 5.51 for the leg, and 4.16 for the arm (van der Zee et al., 1992; Duncan et al., 1995). DPF for cerebral tissue was calculated (DPF = 4.99 + 0.067 × Age^0.814^) according to the manufacturer's guidelines. The tissue saturation index (TSI = Δ[O_2_Hb]/Δ[tHb] × 100%) was calculated from the light attenuation slope along the distance from the three emitting points as detected by the sensor of the receiving optode. Obtained NIRS data were averaged to give values in 1 s intervals, and time-aligned with gas exchange, heart rate and SpO_2_% data. The Δ[O_2_Hb], Δ[HHb] and TSI recordings were presumed to be reliable estimators of changes in tissue deoxygenation status representing regional imbalances between O_2_ delivery and O_2_ utilization in the field of interrogation (Boushel et al., 2001; DeLorey et al., 2003). Total hemoglobin (Δ[tHb]) was analyzed since it reflects changes in tissue blood volume (Boushel et al., 2001; DeLorey et al., 2003).

Values of TSI and Δ[HHb] at peak exercise are referred as TSI_peak_ and Δ_peak_[HHb], respectively. The absolute exercise-induced changes (Δ_t_) in TSI and Δ[HHb] were determined as a subtraction [the value at peak exercise minus the value at baseline walking (5 km · h^−1^)] and are referred as Δ_t_TSI and Δ_t_[HHb], respectively.

#### NIRS inflection point determination

To determine tissue-specific NIRS inflection points (NIP) reflecting local oxygenation changes in leg muscle, arm muscle, and cerebral tissue, the following procedures were performed:

1. Second by second data of Δ[O_2_Hb], TSI, and Δ[HHb] were plotted against time starting from locomotion at 8 km · h^−1^ until the end of exercise. The plottings were performed to all the three tissues of interest (m. vastus lateralis, m. biceps brachii, frontal cerebral cortex) of every subject.
2. The axes and sizes of the plotted figures were scaled to be similar and comparable with each other.
3. Trend lines were drawn and adjusted to describe trends throughout the scatter plots.
4. When there was an angle of = 15° between consecutive trend lines, the intersection between the two trend lines was regarded as an NIP and included in further analyses.
5. A vertical line cutting both the intersection and X-axis (time) was drawn to determine the timing of the inflection point.
6. The pulmonary O_2_ uptake at each inflection point was determined as a 30 s average (time ± 15 s) from second by second data of ${\dot{\text{V}}\text{O}}_{\text{2}}$.

After determining them, the tissue-specific NIP were chosen for further analyses on the following condition; an angle of ≥ 15° between consecutive trend lines had to be observed at the same time point in all the three parameters (Δ[O_2_Hb], TSI, and Δ[HHb]). The change of this magnitude was expected to reflect permanent changes in tissue oxygenation trend. In addition, for cerebral tissue, inflection points with a rise only in cerebral Δ[HHb], were included in further analyses. The tissue-specific inflection points were named as follows: NIP_LegAT_ (the NIP that was observed in the leg muscle and was the closest to AT), NIP_LegRC_, NIP_ArmAT_, NIP_ArmRC_, NIP_CerAT_, and NIP_CerRC_, respectively.

### Blood O_2_ carrying capacity

To quantify blood O_2_ carrying capacity, tHb-mass was determined by the optimized carbon monoxide rebreathing method (SpiCO, BloodTech, Bayreuth, Germany) (Schmidt and Prommer, 2005) on a separate visit. Total Hb-mass was divided by body weight and, thus, handled as relative tHb-mass in analyses. Capillary blood was drawn from a fingertip to analyze hemoglobin concentration and the fraction of carboxyhemoglobin by a blood gas analyzer (ABL725, Radiometer, Copenhagen, Denmark). Peak O_2_ uptake values were then plotted against the tHb-mass values, and a linear regression line was fitted. The slope of this line represented the magnitude of change in ${\dot{\text{V}}\text{O}}_{\text{2peak}}$ vs. a given change in tHb-mass [Δ${\dot{\text{V}}\text{O}}_{\text{2peak}}$ (ml · min^−1^)/ΔtHb-mass (g)].

Relationships between tissue oxygenation and blood O_2_ carrying capacity were also examined: values of Δ[O_2_Hb], TSI, and Δ[HHb] both at each submaximal intensity and peak exercise were plotted against the tHb-mass values, and linear regression lines were fitted.

### Statistical analysis

All parameters are expressed as mean ± standard deviation (SD). The mean values of the last 30 s at rest, during baseline walking, and each step of incremental exercise were chosen for further analyses. Peak O_2_ uptake (${\dot{\text{V}}\text{O}}_{\text{2peak}}$) was determined as the highest value of a 60 s moving average “window”. The NIRS values obtained during the incremental exercise were compared with the values at 5 min during baseline walking (repeated measures ANOVA with Sidak *post-hoc* analysis). The rationale for choosing this approach, instead of comparing values with the values at rest, was that the activation of the muscle pump at the onset of exercise expels blood from muscles toward the heart, which is expected to explain the rapid temporary changes in NIRS measurements (DeLorey et al., 2003). Thus, the NIRS device was zeroed at rest, but the results at 5 min baseline walking were chosen to be a reference point. Temporal coincidence of NIPs and ventilatory thresholds was tested with paired samples *t*-test. Relationships between key parameters were determined by Pearson's correlation coefficient. Statistical significance was defined at the *p* < 0.05 level, and all results were computed with PASW Statistics 18.0 (IBM Corporation, Somers, NY, USA).

## Results

### Cardiopulmonary responses

**Figure 1:**
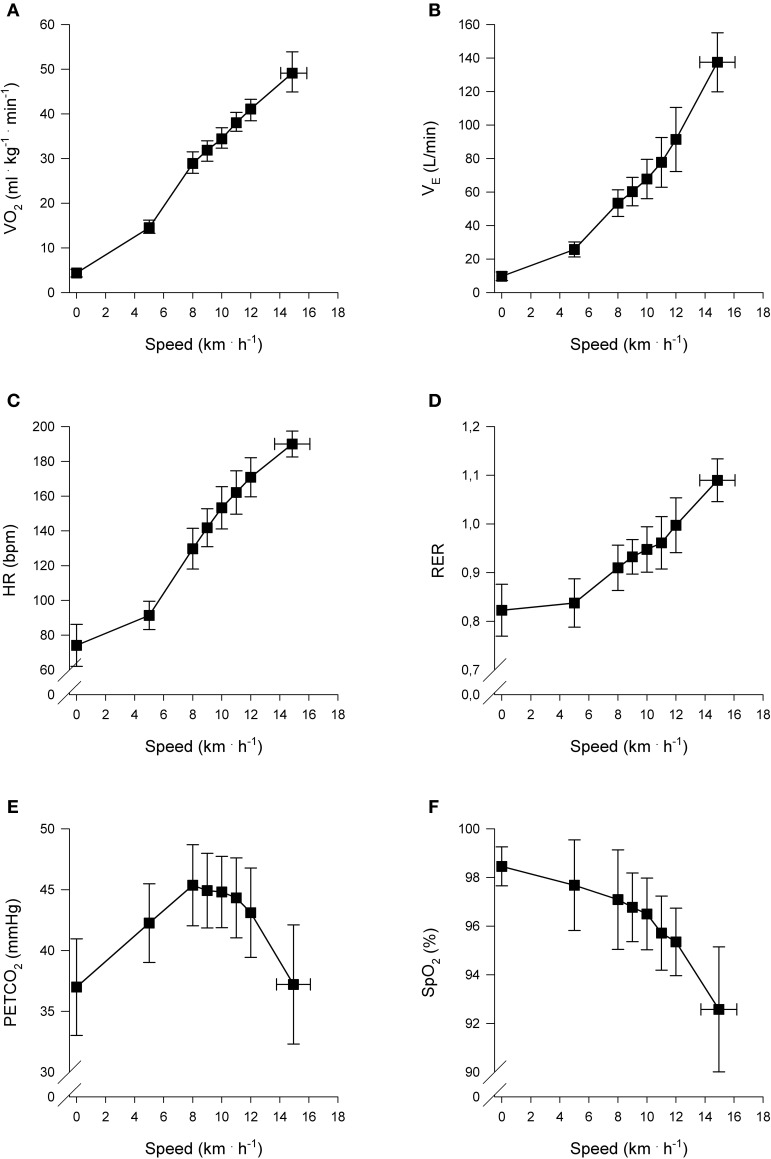
**Cardiopulmonary responses to incremental exercise (*n* = 22).** Pulmonary oxygen uptake (${\dot{\text{V}}\text{O}}_{\text{2}}$) **(A)**, Ventilation (${\dot{\text{V}}}_{\text{E}}$) **(B)**, Heart rate (HR) **(C)**, Respiratory exchange ratio (RER) **(D)**, End-tidal carbon dioxide partial pressure (PETCO_2_) **(E)**, and Arterial oxygen saturation (SpO_2_%) **(F)**, as a function of locomotion speed. Presented speeds include rest, baseline walking, speeds accomplished by each subject and speed at peak exercise.

The cardiopulmonary responses at AT, RC, and peak exercise are presented in Table 2. The mean responses of ${\dot{\text{V}}\text{O}}_{\text{2}}$, ${\dot{\text{V}}}_{\text{E}}$, HR, RER, PETCO_2_-, and SpO_2_ to exercise are presented in Figure 1.

**Table 2:** **Cardiopulmonary data of the subjects at anaerobic threshold, respiratory compensation point, and peak exercise (*n* = 22)**.

	**AT**	**RC**	**Peak exercise**
Speed (km · h^−1^)	9.7 ± 1.4	12.4 ± 1.4	14.9 ± 1.2
${\dot{\text{V}}\text{O}}_{\text{2}}$ (ml · kg^−1^ · min^−1^)	34 ± 5	43 ± 5	50 ± 6
%${\dot{\text{V}}\text{O}}_{\text{2}}\text{R}$	65 ± 7	84 ± 6	–
${\dot{\text{V}}}_{\text{E}}$ (L/min)	64.7 ± 10.8	92.6 ± 14.1	137.5 ± 17.6
HR (bpm)	148 ± 12	173 ± 6	190 ± 7
RER	0.94 ± 0.04	1.01 ± 0.04	1.09 ± 0.04
PETCO_2_ (mmHg)	45.2 ± 3.2	42.8 ± 3.1	37.2 ± 4.9
SpO_2_%	–	–	93 ± 3

Values are means ± SD. AT, anaerobic threshold; RC, respiratory compensation point; ${\dot{\text{V}}\text{O}}_{\text{2}}$, pulmonary oxygen uptake; %${\dot{\text{V}}\text{O}}_{\text{2}}\text{R}$, percentage value of ${\dot{\text{V}}\text{O}}_{\text{2}}$ reserve; ${\dot{\text{V}}}_{\text{E}}$, ventilation; HR, heart rate; RER, respiratory exchange ratio; PETCO_2_, end-tidal carbon dioxide partial pressure; SpO_2_%, arterial oxygen saturation.

### Regional cerebral and muscle oxygenation

#### NIRS measurements

**Figure 2:**
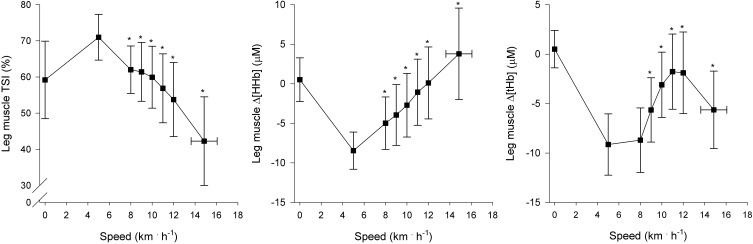
**NIRS recordings from leg muscle (m. vastus lateralis) (*n* = 22).** Tissue saturation index (TSI), and changes in deoxyhemoglobin concentration (Δ[HHb]) and total hemoglobin concentration (Δ[tHb]), as a function of locomotion speed. See details in text and Figure 1. ^*^Significantly different from baseline walking (*p* < 0.05).

Regional muscle and cerebral oxygenation patterns are shown in Figures 2 (leg muscle), 3 (arm muscle), and 4 (cerebral tissue). In the leg muscle (m. vastus lateralis), Δ[O_2_Hb] and TSI decreased (−8.7 ± 5.1 μM; −28.7 ± 10.9%, respectively) from baseline walking to peak exercise, while Δ[HHb] and Δ[tHb] increased (12.2 ± 5.8 μM; 3.5 ± 3.8 μM, respectively). In the arm muscle (m. biceps brachii), Δ[O_2_Hb] and TSI decreased (−19.1 ± 9.0 μM; −42.0 ± 20.7%, respectively), while Δ[HHb] and Δ[tHb] increased (21.9 ± 12.7 μM; 2.8 ± 13.8 μM, respectively). In the cerebral cortex, TSI decreased (−9.2 ± 7.6%), while Δ[O_2_Hb], Δ[HHb], and Δ[tHb] increased (3.5 ± 4.7 μM; 6.2 ± 2.4 μM; 9.8 ± 4.9 μM, respectively). Deoxygenation at peak exercise was greatest in the arm muscle, then in the leg muscle, and least in cerebral tissue as detected by TSI_peak_ (arm vs. leg; arm vs. cerebral; leg vs. cerebral, all *p* < 0.001) and Δ_peak_[HHb] (arm vs. leg, *p* < 0.001; arm vs. cerebral, *p* < 0.01; leg vs. cerebral, n.s.).

**Figure 3:**
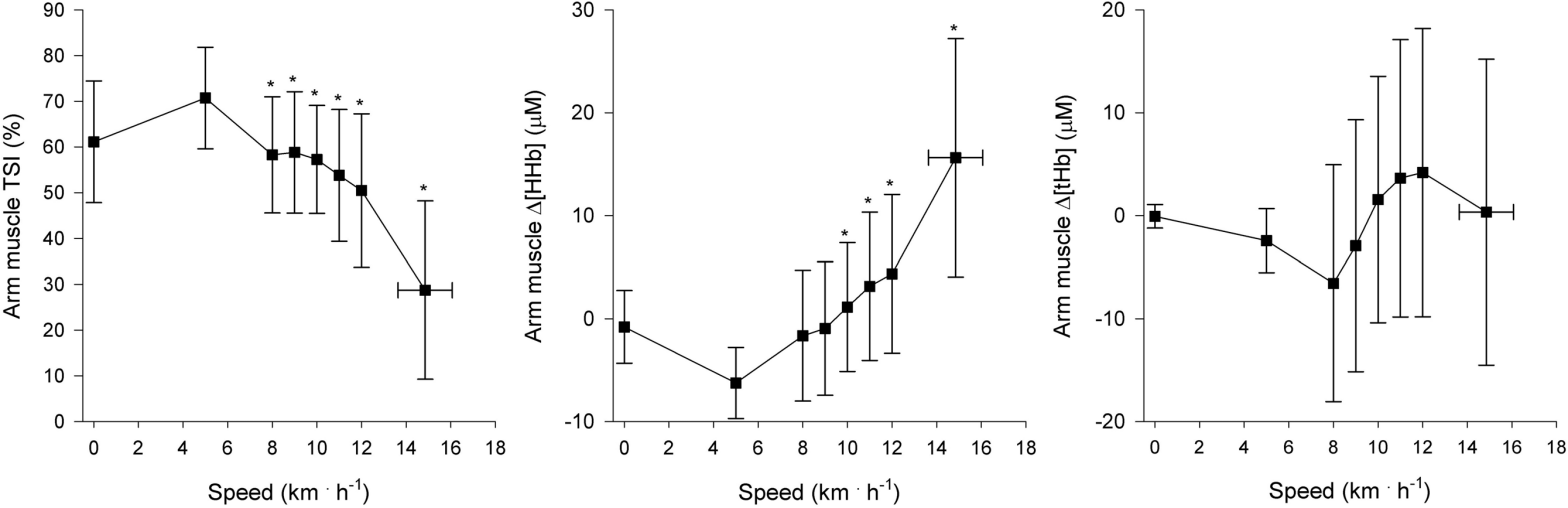
**NIRS recordings from arm muscle (m. biceps brachii) (*n* = 22).** Tissue saturation index (TSI), and changes in deoxyhemoglobin concentration (Δ[HHb]) and total hemoglobin concentration (Δ[tHb]), as a function of locomotion speed. See details in text and Figure 1. ^*^Significantly different from baseline walking (*p* < 0.05).

**Figure 4:**
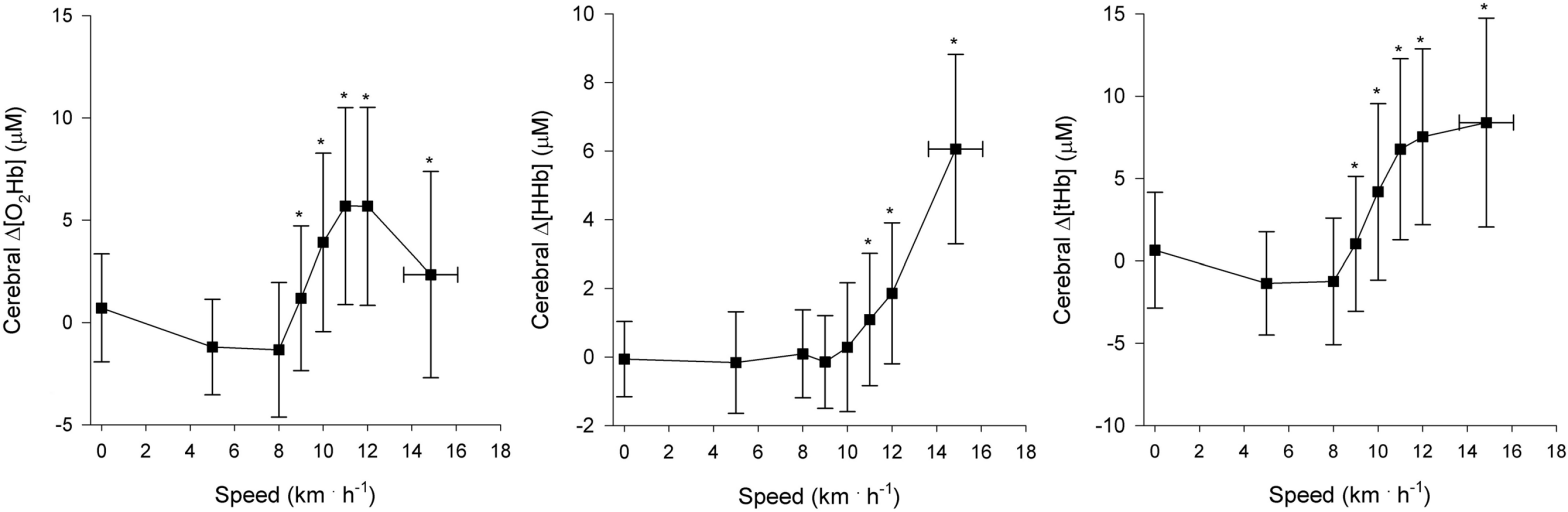
**NIRS recordings from cerebral tissue (frontal cortex) (*n* = 22).** Changes in oxyhemoglobin concentration (Δ[O_2_Hb]), deoxyhemoglobin concentration (Δ[HHb]), and total hemoglobin concentration (Δ[tHb]) as a function of locomotion speed. See details in text and Figure 1. ^*^Significantly different from baseline walking (*p* < 0.05).

In the leg muscle, Δ_t_[HHb] had a positive association with ${\dot{\text{V}}\text{O}}_{\text{2peak}}$ (*r* = 0.48, *p* < 0.05). No other relationships between the extent of leg muscle deoxygenation and ${\dot{\text{V}}\text{O}}_{\text{2peak}}$ were observed at any exercise intensities. In arm muscle- and cerebral tissue, the extent of deoxygenation did not associate with ${\dot{\text{V}}\text{O}}_{\text{2peak}}$.

#### NIRS inflection point determination

**Figure 5:**
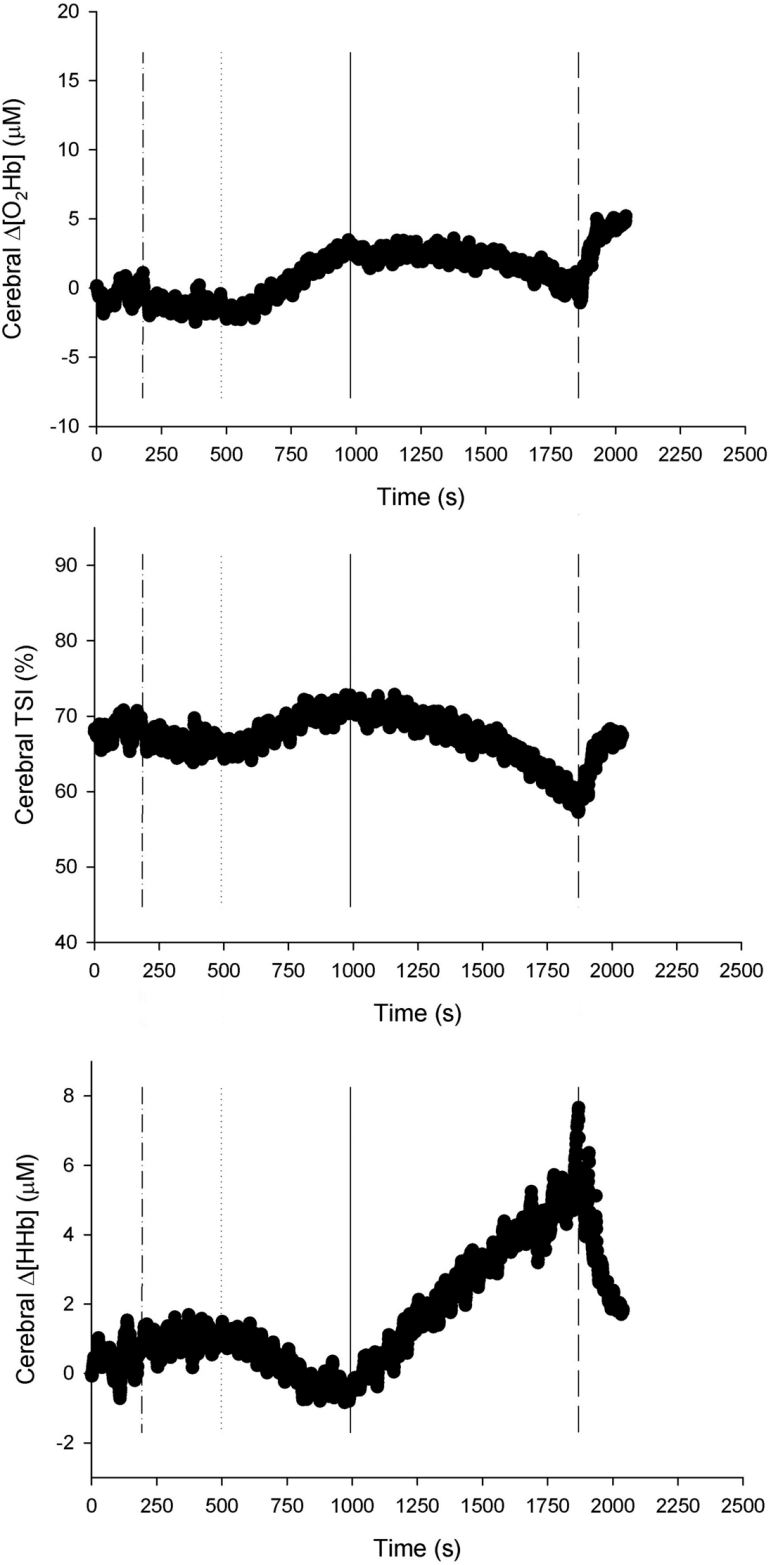
**NIRS recordings from the cerebral tissue (frontal cortex) of a representative subject (*n* = 1).** Changes in oxyhemoglobin concentration (Δ[O_2_Hb]), tissue saturation index (TSI), and changes in deoxyhemoglobin concentration (Δ[HHb]), as a function of time. Baseline walking 5 km · h^−1^ begins at time of 180 s (dash-dot line). In this case, one NIRS inflection point (solid line) has been found between the beginning of locomotion at 8 km · h^−1^ (dotted line) and the end of exercise (dashed line).

An example of NIP determination of the cerebral tissue is presented in Figure 5. For the 22 subjects, a total of 24 NIPs were observed in the leg muscle, 36 in the arm muscle, and 64 in the cerebral tissue. That is to say, a per-person average of 1.1 NIPs were observed in the leg muscle, 1.6 in the arm muscle, and 2.9 in the cerebral tissue. Of the 24 NIPs observed in the leg muscle, 16 reflected deoxygenation (Δ[O_2_Hb]↓, TSI↓ and Δ[HHb]↑), 1 was indefinable (e.g., Δ[O_2_Hb]↓, Δ[HHb]↓), and 7 reflected oxygenation (Δ[O_2_Hb]↑, TSI↑ and Δ[HHb]↓). In the arm muscle, 30 NIPs reflected deoxygenation and 6 reflected oxygenation. In cerebral tissue, 63 NIPs reflected deoxygenation, while 1 was indefinable. The data of six tissue-specific NIPs being the closest to the ventilatory thresholds are presented in Table 3. There were no differences between the NIPs closest to AT and AT (NIP_LegAT_ vs. AT, *p* = 0.587; NIP_ArmAT_ vs. AT, *p* = 0.356; NIP_CerAT_ vs. AT, *p* = 0.332). Concerning the NIPs closest to RC, NIP_LegRC_ did not differ from RC (NIP_LegRC_ vs. RC, *p* = 0.058), while NIP_ArmRC_ (vs. RC, *p* < 0.001) and NIP_CerRC_ (vs. RC, *p* < 0.05) did.

**Table 3:** **Data of the six tissue-specific NIRS inflection points (NIP) being the closest to the ventilatory thresholds (anaerobic threshold and respiratory compensation point)**.

	***n***	**${\dot{\text{V}}\text{O}}_{\text{2}}$ (ml · kg^−1^ · min^−1^)**		***n***	**${\dot{\text{V}}\text{O}}_{\text{2}}$ (ml · kg^−1^ · min^−1^)**
AT	22	34 ± 5	RC	22	43 ± 5
NIP_LegAT_	9	34 ± 5	NIP_LegRC_	9	46 ± 6
NIP_ArmAT_	6	33 ± 3	NIP_ArmRC_	17	46 ± 6^***^
NIP_CerAT_	21	33 ± 4	NIP_CerRC_	19	45 ± 7^*^

Values are means ± SD. n, number; ${\dot{\text{V}}\text{O}}_{\text{2}}$, pulmonary oxygen uptake; AT, anaerobic threshold; RC, respiratory compensation point; NIP_LegAT_, NIRS inflection point that is observed in the leg muscle and is the closest to AT. The other five NIRS inflection points were determined according to the same logic as NIP_LegAT_. Significantly different from the RC (

* p < 0.05;

*** p < 0.001).

NIPs that reflected deoxygenation (Δ[O_2_Hb]↓, TSI↓ and Δ[HHb]↑) had consistently positive associations with ${\dot{\text{V}}\text{O}}_{\text{2peak}}$ when examining absolute ${\dot{\text{V}}\text{O}}_{\text{2}}$ at NIPs (e.g., ${\dot{\text{V}}\text{O}}_{\text{2}}$ at the second NIP reflecting deoxygenation in m. vastus lateralis vs. ${\dot{\text{V}}\text{O}}_{\text{2peak}}$: *r* = 0.85, *p* < 0.05). However, when examining %${\dot{\text{V}}\text{O}}_{\text{2}}\text{R}$ at NIPs, no associations between NIPs and ${\dot{\text{V}}\text{O}}_{\text{2peak}}$ were observed.

The NIP reflecting acceleration of arm deoxygenation was observed in 10 subjects during last two minutes of exercise (63 ± 42 s before exhaustion). In nine of those 10 subjects, an accelerated rise of ${\dot{\text{V}}}_{\text{E}}$ occurring 60 ± 72 s before the acceleration of arm deoxygenation was also observed.

### Blood O_2_ carrying capacity

Total Hb-mass of the subjects was 928 ± 105 g (11.6 ± 1.2 g · kg^−1^), and it had a positive association with ${\dot{\text{V}}\text{O}}_{\text{2peak}}$ (*r* = 0.81, *p* < 0.001). When individual ${\dot{\text{V}}\text{O}}_{\text{2peak}}$ values were plotted against individual tHb-mass, and a linear regression line [Δ${\dot{\text{V}}\text{O}}_{\text{2peak}}$ (ml · min^−1^)/ΔtHb-mass (g)] was fitted, a 1 g change in tHb-mass was associated with a 3.6 ml · min^−1^ change in ${\dot{\text{V}}\text{O}}_{\text{2peak}}$.

**Figure 6:**
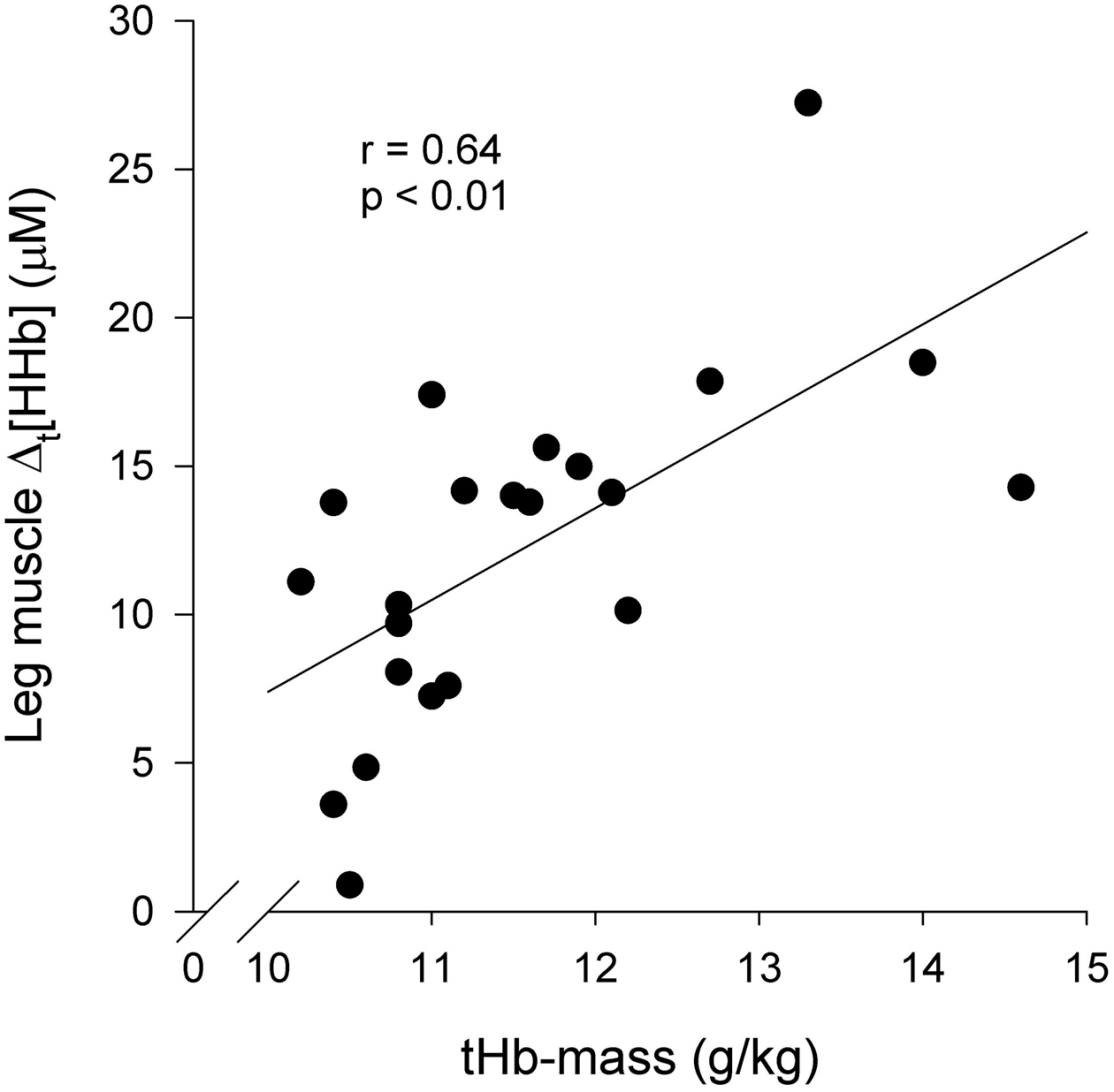
**Association between blood O_2_ carrying capacity and leg muscle (m. vastus lateralis) deoxygenation at peak exercise (*n* = 22): Δ_t_[HHb] vs. tHb-mass.** Δ_t_[HHb] = Δ[HHb] at peak exercise—Δ[HHb] during baseline walking 5 km · h^−1^; tHb-mass = total hemoglobin mass.

Figure 6 presents an association between blood O_2_ carrying capacity and the extent of leg muscle deoxygenation at peak exercise. Altogether, the following associations were observed: tHb-mass and Δ_t_[HHb] (*r* = 0.64, *p* < 0.01), tHb-mass and TSI_peak_ (*r* = −0.50, *p* < 0.05), and tHb-mass and Δ_peak_[HHb] (*r* = 0.46, *p* < 0.05). On the contrary, the extent of arm muscle- or cerebral tissue deoxygenation had no consistent associations with blood O_2_ carrying capacity; nor were there consistent associations between blood O_2_ carrying capacity and the extent of tissue deoxygenation during any submaximal intensities.

## Discussion

This is the first study to measure alveolar gas exchange and tissue oxygenation in highly active leg muscle and less active arm muscle, as well as in the cerebral cortex, simultaneously during incremental treadmill exercise, and to seek, if blood O_2_ carrying capacity affects tissue oxygenation. A novel method was employed to determine NIP. Regional oxygenation of different tissues was characterized by NIPs, and comparing the timings of the tissue-specific NIPs with alterations in alveolar gas exchange showed that tissue oxygenation during incremental treadmill exercise is consistent with previous findings made during incremental cycling. Another novel finding is that subjects with high ${\dot{\text{V}}\text{O}}_{\text{2peak}}$ and tHb-mass experienced greater leg muscle deoxygenation (as analyzed by an increase in Δ[HHb]) at peak exercise than their counterparts with lower ${\dot{\text{V}}\text{O}}_{\text{2peak}}$ and blood O_2_ carrying capacity. The implications of the findings will be discussed in depth.

### Cardiopulmonary responses

The cardiopulmonary responses observed in this study are consistent with previous reports on healthy subjects. The subjects had a mean ${\dot{\text{V}}\text{O}}_{\text{2peak}}$ of 50 ml · kg^−1^ · min^−1^, representing good gender- and age-related fitness status (Shvartz and Reibold, 1990).

### Regional cerebral and muscle oxygenation

NIRS was used at multiple sites (m. vastus lateralis, m. biceps brachii, and frontal cerebral cortex) to provide information pertaining to the regional changes in oxygenation in these tissues during incremental treadmill exercise. Tissue oxygenation results from arterial O_2_ saturation (SpO_2_%), local metabolic rate, and tissue blood flow (Lee et al., 2011). In this study, SpO_2_% remained relatively constant at 95–100% during submaximal intensities, and was 93% on average at peak exercise, which indicates mild-to-moderate exercise-induced arterial hypoxemia (Dempsey and Wagner, 1999). Consequently, it is expected that changes observed in tissue oxygenation are mainly the result of increased local O_2_ extraction and utilization due to increasing tissue-specific metabolic demand and alterations in blood volume and/or flow.

#### Leg muscle oxygenation

In the leg muscle (m. vastus lateralis), the oxygenation pattern is in accordance with other studies that have examined incremental treadmill exercise (Hiroyuki et al., 2002; Lee et al., 2011). At the onset of baseline walking, TSI immediately increased above standing level, which indicates increased leg muscle oxygenation. The rapid increase of TSI resulted from activation of the muscle pump, that expelled pooling venous blood toward the heart (DeLorey et al., 2003), and thus, led to decreased Δ[tHb]. At speeds of greater than or equal to 8 km · h^−1^, subjects began running and leg muscle oxygenation decreased (TSI↓, Δ[HHb]↑) with the increase in running speed and O_2_ demand.

At the speeds of greater than or equal to 8 km · h^−1^, Δ[tHb] increased, reflecting an increase in leg muscle blood volume. However, in agreement with previous findings (Legrand et al., 2007), Δ[tHb] decreased at high intensities (≥ 12 km · h^−1^) but remained significantly higher than values observed during baseline walking. The decrease primarily resulted from local vasoconstriction (Secher and Volianitis, 2006) and possibly from mechanical constraints (Kowalchuk et al., 2002). Moreover, blood flow distribution changes (i.e., blood flow heterogeneity decreases) within the active muscle mass with increasing speed (Heinonen et al., 2007). Further investigations are needed to determine the combined contribution of these factors to changes in muscle blood volume during high intensities.

In this study, we employed a novel method to determine NIP. In the leg muscle, an average of 1.1 NIPs per subject were observed; however, there was inter-individual variability. The main findings concerning NIP determination are: (1) NIPs observed coincided with AT and/or RC; (2) both NIP_LegAT_ and NIP_LegRC_ were manifested in nine subjects; (3) NIPs reflecting deoxygenation had positive associations with ${\dot{\text{V}}\text{O}}_{\text{2peak}}$ when examining absolute ${\dot{\text{V}}\text{O}}_{\text{2}}$ at NIPs, but when examining relative values (%${\dot{\text{V}}\text{O}}_{\text{2}}\text{R}$), no associations were observed. The 1st and 2nd findings imply that metabolic changes within the muscle tissue affect oxygenation changes, and are one essential part of ventilatory response control during incremental exercise (Turner, 1991; Bhambhani, 2004), but there likely is more inter-individual variability during treadmill exercise than during cycling. It should be noted that NIP_LegRC_ was observed slightly later than RC, although the difference was not statistically significant. The 3rd finding is congruent with the previously reported rightward shift of the Δ[HHb] pattern relative to absolute exercise capacity in individuals of higher aerobic capacity (Boone et al., 2009). However, it is incongruent with a rightward shift of the Δ[HHb] pattern relative to percentage of exercise capacity in individuals with higher ${\dot{\text{V}}\text{O}}_{\text{2peak}}$, also reported by Boone et al. (2009). These data suggest that matching local muscle perfusion to ${\dot{\text{V}}\text{O}}_{\text{2}}$ at low exercise intensities is likely to be better in subjects with higher aerobic capacity. Similar observations have been made during static knee-extensor exercise (Kalliokoski et al., 2005). Thus, subjects with lower ${\dot{\text{V}}\text{O}}_{\text{2peak}}$ place greater reliance on O_2_ extraction in providing adequate ${\dot{\text{V}}\text{O}}_{\text{2}}$, which highlights their lower O_2_ delivery (Bassett and Howley, 2000).

#### Arm muscle oxygenation

This is the first study to examine arm muscle oxygenation during incremental treadmill exercise. The shape of the arm muscle (m. biceps brachii) oxygenation pattern resembles that of the leg muscle. At the onset of baseline walking, arm muscle oxygenation rapidly increased and then remained relatively constant. Thereafter at the speeds of greater than or equal to 8 km · h^−1^, an initial moderate decrease of oxygenation level was observed, followed by a rapid decrease during more severe exercise. At peak exercise, deoxygenation was greatest in the arm muscle in comparison with the leg muscle and cerebral tissue. These findings are consistent with previous findings made during incremental cycling (Ogata et al., 2007; Peltonen et al., 2012).

An average of 1.6 NIPs per subject with inter-individual variability were observed in the arm muscle. Concerning the timings of the NIPs, NIP_ArmAT_ coincided with AT but was manifested only in six subjects, suggesting that arm muscle deoxygenation hardly couples with ventilatory responses at low intensities during incremental treadmill exercise. However, NIP_ArmRC_ did not coincide with RC but was observed later than RC. In addition, a NIP reflecting acceleration of arm deoxygenation was observed in 10 subjects during the last two minutes of exercise, and an accelerated rise of ${\dot{\text{V}}}_{\text{E}}$ occurred in nine of those 10 subjects approximately 1 min before the deoxygenation acceleration. Ogata et al. (2007) and Peltonen et al. (2012) have similarly reported that the magnitude of decrease in O_2_ delivery to less active muscle is coupled to the magnitude of increase in the amount of hyperventilation during incremental cycling. Ogata et al. (2007) suggested that the association would result from metabolite accumulation and the concomitant metabolic acidosis, which concurrently affect both hyperventilation and O_2_ delivery to inactive muscle. The acceleration of arm deoxygenation might also be explained by exercise-induced sympathetic flow and the consequential vasoconstriction to support the prevailing blood pressure, and thus favoring blood flow to working muscles (Secher and Volianitis, 2006).

#### Cerebral oxygenation

Rooks et al. (2010) recently described that cerebral tissue oxygenation has a quadratic response to incremental exercise: it increases from low-to-hard intensities, which is followed by a plateau or decline toward baseline at very hard, maximal intensities. They specified that in aerobically trained people, the plateau or a slight decline in cerebral Δ[O_2_Hb] concurrent with increasing Δ[HHb] was observed at exhaustive, maximal intensities. Our findings are similar: Δ[O_2_Hb] rose from low-to-hard intensities and fell slightly at the speeds of greater than 12 km · h^−1^, whereas Δ[HHb] increased along with running speed.

Thus, cerebral deoxygenation was manifested, and it accelerated after RC, because RC was followed by NIP_CerRC_. We measured PETCO_2_ [an indirect estimate of partial pressure of arterial CO_2_ (PaCO_2_)], which began to decrease systematically at RC due to hyperventilation as reported previously (Beaver et al., 1986). The decrease in PaCO_2_ is known to lead to cerebral vasoconstriction (Ogoh and Ainslie, 2009). However, no significant cerebral vasoconstriction was observed in our study, demonstrated by the plateau of Δ[tHb] level at high intensities. Previous studies (Subudhi et al., 2007; Rupp and Perrey, 2008) have also reported cerebral Δ[tHb] level remaining elevated in trained individuals at peak exercise, whereas Bhambhani et al. (2007) reported a post-RC decrease in cerebral blood volume (Δ[tHb]) in individuals with a mean ${\dot{\text{V}}\text{O}}_{\text{2peak}}$ of <40 ml · kg^−1^ · min^−1^. One reason for the variability of Δ[tHb] levels at peak exercise may be lower peripheral chemosensitivity and lower submaximal ${\dot{\text{V}}}_{\text{E}}$ of trained individuals, which attenuate PaCO_2_ reduction at high intensities and result in less cerebral vasoconstriction (Weil and Swanson, 1991).

In cerebral tissue, an average of 2.9 NIPs per subject were observed with inter-individual variability; the number is higher than in muscle tissues because NIPs reflecting the acceleration of cerebral deoxygenation as a rise in cerebral Δ[HHb] only were also included in analyses. Oxygen uptake at NIPs reflecting cerebral deoxygenation correlated positively with ${\dot{\text{V}}\text{O}}_{\text{2peak}}$, implying that cerebral O_2_ delivery, in relation to O_2_ utilization, decreases later in trained individuals throughout incremental exercise. However, cerebral O_2_ delivery stays adequate until exhaustion and does not limit exercise performance during normoxia (Subudhi et al., 2007).

### Blood O_2_ carrying capacity

Peak O_2_ uptake is a key determinant of endurance performance. Indispensable prerequisites for high ${\dot{\text{V}}\text{O}}_{\text{2peak}}$ include high tHb-mass, which is determining blood O_2_ carrying capacity, and thus, is a vital part of O_2_ delivery (Schmidt and Prommer, 2010). The tHb-mass had a high correlation with ${\dot{\text{V}}\text{O}}_{\text{2peak}}$ also in our study. Additionally, we showed that a 1 g change in tHb-mass was associated with a 3.6 ml · min^−1^ change in ${\dot{\text{V}}\text{O}}_{\text{2peak}}$, which is similar to the previous value (1 g, ~4 ml · min^−1^) (Schmidt and Prommer, 2010).

The association between blood O_2_ carrying capacity and the extent of leg muscle deoxygenation at peak exercise was observed: the higher the O_2_ carrying capacity, the greater leg muscle deoxygenation at peak exercise. This can be explained by several peripheral adaptations induced by endurance training. First, the capillary density of skeletal muscle is increased, increasing the surface available for blood-tissue exchange (Brodal et al., 1977). Consequently, the surface area for O_2_ diffusion increases, the average diffusion path length within the muscle decreases, and the length of time for diffusive exchange between blood and tissue increases (Kalliokoski et al., 2001; Prior et al., 2004). The diffusion rate of O_2_ across myocyte membranes is limited, however, at exercise intensities over 50% of the maximal work rate (Richardson et al., 2001). Second, mitochondrial density is elevated (Holloszy and Coyle, 1984). Third, the activity of oxidative enzymes is enhanced (Holloszy and Coyle, 1984). Consequently, we hypothesize that the preceding adaptations to endurance training enable the attainment of higher deoxygenation level at peak exercise in individuals with higher capacity for O_2_ carrying and delivery. On the contrary, the extent of arm muscle- or cerebral tissue deoxygenation had no consistent associations with blood O_2_ carrying capacity.

We initially hypothesized that at peak exercise, blood O_2_ carrying capacity and ${\dot{\text{V}}\text{O}}_{\text{2peak}}$ would have no association with the extent of tissue deoxygenation in muscle, because Hogan et al. (1999) had reported a similar intracellular metabolic environment attained at exhaustion among the varied fraction of inspired O_2_. In earlier studies concerning the association between ${\dot{\text{V}}\text{O}}_{\text{2peak}}$ and deoxygenation (Bae et al., 1996; Neary et al., 2002; Boone et al., 2009), conflicting results had been detected. Thus, further studies are warranted.

### Methodological considerations

The strengths of NIRS method are ease of application, non-invasiveness, potential for providing temporal resolution, and low signal noise during exercise (Boushel et al., 2001). The main weakness is that NIRS, as used in this study, provides a quantitative measure of regional blood volume, not blood flow. Thus, we were unable to draw extensive conclusions concerning blood flow and, in larger scale, O_2_ delivery. Concerning the contribution of muscle myoglobin (Mb) to the NIRS signal, it was expected that Hb is predominantly reflected in human muscle studies (Ferreira et al., 2007b), although NIRS cannot measure Hb and Mb separately. Subcutaneous fat tissue was not taken into account in NIRS analyses, because the standard deviation of fat % was relatively small.

The optical transport coefficients used in NIRS methods are subject to inter-individual variability during exercise, particularly when wavelength of 690 nm is used (Ferreira et al., 2007a). However, when wavelengths between 760 and 900 nm are used in adult humans, all the three tissues of our interest exhibit a broadly similar and comparatively weak wavelength dependence of optical transport coefficients (Matcher et al., 1997) and are not affected by exercise (Ferreira et al., 2007a). Thus, the wavelengths used in this study (765 and 860 nm) were expected to provide data that is reliable and comparable between subjects.

During treadmill exercise, different locomotion strategies (e.g., stride frequency, stride length) and variations in anthropometrics (e.g., body weight, limb lengths) across the subjects most likely affected muscle recruitment patterns, levels of muscle work, and local cardiovascular responses. Varying running mechanics across subjects may also explain the inter-individual variability of oxygenation responses observed in our subjects, also noted by previous investigators (e.g., Lee et al., 2011), and reflects the variability of metabolic efficiency of running, which may vary as much as 40% across individuals (Joyner and Coyle, 2008). One must also note that we only measured m. vastus lateralis and m. biceps brachii, while there is heterogeneity in activation and metabolism within muscle groups during exercise (Kalliokoski et al., 2011).

## Conclusions

This was the first study to measure alveolar gas exchange and tissue oxygenation in leg and arm muscle, as well as in cerebral cortex, simultaneously during incremental treadmill exercise. This was also the first study to examine associations between blood O_2_ carrying capacity and tissue oxygenation. We demonstrated that regional tissue oxygenation is characterized by tissue-specific inflection points, and tissue oxygenation in relation to alveolar gas exchange during incremental treadmill exercise resembles that during incremental cycling. It was also found out, that O_2_ delivery to less active m. biceps brachii may be limited by an accelerated increase in ventilation at high running intensities. Another novel finding was that a greater capacity for blood O_2_ carrying is associated with greater level of highly active m. vastus lateralis deoxygenation at peak treadmill exercise.

### Conflict of interest statement

The authors declare that the research was conducted in the absence of any commercial or financial relationships that could be construed as a potential conflict of interest.
